# Accidental Proving of Carbolic Acid

**Published:** 1893-11

**Authors:** J. C. Fahnstock


### ACCIDENTAL PROVING OF CARBOLIC ACID.

J. C. Fahnstock, M. D.

Mrs. R. æt, thirty-two, dark hair, nervous temperament, and ordinarily enjoys fair health.

On the morning of July 22d, 1888, was washing, and she, in order to remove some "rust stains" in a garment, put what she supposed to be Carbolic Acid, diluted one to twenty in a little hot water, and began to rub the clothes, inhaling the steam with the acid; but it was pure Carbolic Acid instead of diluted.

In about ten minutes she began to feel very queer, and being alone, she started to run across the street to one of her neighbors, but before reaching the house she fell prostrate, pale and gasping for breath. She was taken into the house, and I was called in haste. On my arrival I found my patient propped up with pillows, being unable to lie down, continually gasping for breath, with a trembling all over, so much that she wanted her hands held; "pricking like needles all over her body;" unable to raise the right arm.

Pale face, dilated pupils, cold hands and feet.

Thirst, wanted a drink of water every few minutes.

In about half an hour, nausea, but no vomiting.

Pain in lumbar region.

These symptoms continued about four hours, but gradually getting less and less, and finally disappeared in the evening, not leaving any bad effects the next day, and she again enjoys her usual good health.

I report this accidental proving of Carbolic Acid thinking it might prove to be some benefit to some suffering one, as I was forcibly struck with the great similarity of symptoms to asthma.

There is an accidental proving of Carbolic Acid by T. D. Pritchard, reported in the transactions of the Homœopathic Medical Society of New York, new series. In his concluding remarks he says : "For the last year I have been much troubled with shortness of breath on going up-hill or up-stairs, or when running, without cough. Since I recovered from the effects of the Carbolic Acid I am almost entirely free from the shortness of breath.—*The Homœopathic News*, vol. XXII, July, 1893.

